# Switching from adalimumab originator to biosimilars in hidradenitis suppurativa: What's beyond cost‐effectiveness?

**DOI:** 10.1111/dth.15803

**Published:** 2022-09-14

**Authors:** Gabriele Roccuzzo, Giulia Rozzo, Lorenza Burzi, Federica Repetto, Paolo Dapavo, Simone Ribero, Pietro Quaglino

**Affiliations:** ^1^ Department of Medical Sciences Section of Dermatology, University of Turin Turin Italy

**Keywords:** adalimumab, biosimilar, hidradenitis Suppurativa, psoriasis

## Abstract

In the last years, adalimumab biosimilars have represented a commonly used alternative to the originator agent in the treatment of moderate to severe hidradenitis suppurativa. As of today, studies investigating the switch from adalimumab originator to biosimilar, following pharmacoeconomic policies, are lacking. Herein we present a real‐life setting retrospective study aimed at assessing the safety and efficacy of this switch in 37 patients, evaluated for 12 months in terms of IHS4 (International Hidradenitis Suppurativa Severity Score System) and HiSCR (Hidradenitis Suppurativa Clinical Response). Overall, no significant differences emerge between adalimumab originator and biosimilar in terms of clinical response following non‐medical switch. High discontinuation rates (43.2%) raise questions on patients' compliance to the new drug regimen, as severe pain at the injection site represents a substantial cause of biosimilar discontinuation (i.e., 31.5% of the cases). In selected cases, rechallenge with adalimumab originator may represent a valid option, as 66.6% (*n* = 8) of the patients who switched back to the former agent have benefited in terms of tolerability and efficacy. Carefully integrating pharmacoeconomic policies with a thorough assessment regarding the benefit–risk ratio of a nonmedical switch from originator to biosimilars remains essential to provide each HS patient with the best therapeutic option.

## 1 SHORT REPORT

Since their commercialization in 2018, adalimumab biosimilars have represented an accessible alternative to the originator Humira (ABBVIE)™, thanks to their positive impact on health care budgets.^1^ These anti‐TNF‐alpha drugs represent the only biologic agents currently approved for moderate to severe hidradenitis suppurativa (HS). Though biosimilars are validated according to the same standards of pharmaceutical quality, safety, and efficacy that apply to the reference medicine, their performance may differ from the originator, due to different manufacturing processes, excipients, and packaging.^2^ To date, studies investigating the switch from adalimumab originator to biosimilars in HS patients are still lacking, as only few reports have addressed this issue.^3^, ^4^ Herein, we describe the results of a retrospective study carried out in a real‐life setting at the Dermatology clinic of the University of Turin, between December 2018 and December 2021, aimed at evaluating the safety and efficacy of the switch from adalimumab originator to adalimumab biosimilars in HS patients. The study was conducted in accordance with the declaration of Helsinki. Inclusion criteria were: age ≥ 18, Hurley grade ≥2, non‐medical reason for switch (i.e., according to the national health service policies on prescription), standard dose regimen for HS (i.e., 160 mg at day 1, 80 mg at day 15 and 40 mg once a week from day 29). Patients were evaluated every 12 weeks according to IHS4 (International Hidradenitis Suppurativa Severity Score System) and HiSCR (Hidradenitis Suppurativa Clinical Response).^5^, ^6^ Mann‐Whitney and McNemar's tests with Yates correction were used to analyze continuous and paired nominal data, respectively. Patients' characteristics are summarized in Table 1. All patients received at least one conventional treatment before adalimumab (median number of previous lines of therapy of 3, range 1–6), with tetracycline as the most common (64.9% of patients). At adalimumab originator first prescription (T0), 78.4% of the patients were classified as Hurley 2 and 21.6% as Hurley 3, with a mean basal IHS4 of 16.51 ± 9.6. No patient discontinued adalimumab originator since first prescription, remaining an average of 52.7 ± 30.4 weeks on therapy. All 37 patients were subsequently switched to the biosimilar following local pharmacoeconomic policies. At the time of drug switch (Ts), mean ISH4 had improved to 6.6 ± 7.2 (*p* < 0.00001). All patients reached the 3‐month analysis after the switch (T3) and no significant difference was observed compared to Ts in terms of ISH4 (*p* = 0.81). During the year of follow up, 16 patients (43.2%) discontinued treatment (8 due to treatment inefficacy, 5 due to severe injection site pain and 3 for unspecified reasons). The overall mean time on biosimilar was 44.4 ± 17.6 weeks. No severe side effects were observed. The analysis between Ts and last biosimilar administration showed no substantial differences as for ISH4 improving (*p* = 0.64552) nor HiSCR achievement rates (*p* = 0.50233). In the subset of patients who discontinued the biosimilar due to inefficacy, cutaneous worsening was represented by higher mean ISH4 (i.e., 8.75 ± 8.22 at T3 and 14.86 ± 7.97 at time of discontinuation), yet without achieving statistical significance (*p* = 0.12852). Out of the 16 patients who discontinued the biosimilar, 12 were subsequently switched back to adalimumab originator. Currently, 8 patients are still receiving beneficial treatment with the originator, while 5 have been lost to follow up and 3 have been switched to off‐label anti IL17 due to poor disease control, in accordance with literature evidence.^7^ At last, the comparison between the ongoing therapy subgroup (*n* = 21) and the subset of patients who discontinued treatment (*n* = 16) did not reveal any statistically relevant predictors of treatment continuation relating to ISH4 at T0 (*p* = 0.36812), Ts (*p* = 0.38978), and T3 (*p* = 0.4009). Our study collects the largest cohort and the longest follow up ever reported for HS patients treated with adalimumab biosimilars to date, with switching failure rates in line with those previously described.^2^, ^8^ While no significant differences emerge between adalimumab originator and biosimilar in terms of clinical response following nonmedical switch, high discontinuation rates (43.2%) raise questions on patients' compliance to the new drug regimen, since severe pain at the injection site still represents a substantial cause of biosimilar discontinuation (i.e., 31.5% of the cases), as reported by Montero‐Vilchez et al.^2^ In selected cases, rechallenge with adalimumab originator may represent a valid option, as 66.6% (*n* = 8) of our patients who switched back to the former agent have benefited in terms of tolerability and efficacy, with ISH4 improvement from 14.25 ± 8.91 to 9.5 ± 8.94 (*p* = 0.37346). Currently no conclusion can be drawn with certainty as for the underlining factors contributing to the improvement of patients' clinical response and compliance after switching back to adalimumab originator, yet all the characteristics, such as packaging, formulation, and excipients, which are unique for each drug, are likely to have played a role.^2^ Larger studies with higher statistical power are needed to confirm the evidence displayed in this preliminary study. Meanwhile, carefully integrating pharmacoeconomic policies with a thorough assessment regarding the benefit–risk ratio of a nonmedical switch from originator to biosimilars remains essential to provide each HS patient with the best therapeutic option.

**TABLE 1:** Patients' demographics, characteristics, and responses at different times

		*n* (%)	
Total patients		37 (100%)	
Gender (male)		20 (54%)	
Family history positive for HS		8 (21.6%)	
Smokers		24 (65%)	
Age of HS onset (mean ± SD*)*		23.65 ± 9.76	
Age of HS diagnosis (mean ± SD*)*		34.30 ± 13.66	
BMI class (median, range)		27.5 (18.5–39.9)	
Number of prior lines of treatment received (median, range)		3 (1–6)	
Age at Adalimumab starting (mean ± SD)		37.1 ± 13.6	
*Adalimumab starting time* (T0) (*n* = 37)			
Hurley score		Hurley 1: 0 (0%) Hurley 2: 29 (78.4%) Hurley 3: 8 (21.6%)	
HIS4 score		Mean: 16.51 ± 9.6 Median: 14.0	
Number of weeks on originator therapy (mean ± SD)		52.7 ± 30.4	
*Switch to biosimilar* (Ts) (*n* = 37)			
Hurley score		Hurley 1: 4 (10.8%) Hurley 2: 28 (75.7%) Hurley 3: 5 (13.5%)	
IHS4 score		Mean: 6.6 ± 7.2 (p < 0.00001) Median: 5.0	
HiSCR achievement		Achieved: 18 (48.6%) Not achieved: 19 (51.4%)	
3 *months after switch to biosimilar* T3 (*n* = 37)			
Treatment discontinuation		0 (0%)	
HIS4 score (T3)		Mean: 7.4 ± 7.7 (*p* = 0.81) Median: 5.0	
HiSCR achievement (T3)		Achieved: 17 (45.9%) Not achieved: 20 (54.1%)	
6 *months after switch to biosimilar* T6 (*n* = 31)			
Treatment discontinuation		6 (16.2%)	
IHS4 score (T6)		Mean: 6.0 ± 5.6 Median: 6.0	
HiSCR achievement (T6)		Achieved: 16 (43.2%) Not achieved: 21 (56.8%)	
9 *months after switch to biosimilar* T9 (*n* = 26)			
Treatment discontinuation		11 (29.7%)	
HIS4 score (T9)		Mean: 6.9 ± 6.5 Median: 6.0	
HiSCR achievement (T9)		Achieved: 12 (32.4%) Not achieved: 25 (67.6%)	
12 *months after switch to biosimilar* T12 (*n* = 21)			
Treatment discontinuation		16 (43.2%)	
IHS4 score (T12)		Mean: 4.3 ± 4.3 Median: 2.0	
HiSCR achievement (T12)		Achieved: 11 (29.7%) Not achieved: 26 (70.3%)	
Number of weeks on biosimilar therapy (mean ± SD)		44.4 ± 17.6	
	*On therapy at* T12 (*n* = 21)	*Therapy discontinuation at* T12 (*n* = 16)	
HIS4 score T0 (Mean ± SD)	15.76 ± 10.33	17.50 ± 8.77	*p* = 0.36812
HIS4 score Ts (Mean ± SD)	6.0 ± 7.22	7.44 ± 7.33	*p* = 0.38978
HIS4 score T3 (Mean ± SD)	5.9 ± 5.00	9.4 ± 5.50	*p* = 0.4009

*Note*: Hurley score (1: inflammatory nodule or abscess, single or multiple, without sinus tracts and scarring; 2: single or multiple widely separated abscesses and nodules with sinus tract formation or scarring; 3: multiple interconnected sinus tracts, scarring, and abscesses across entire score). HIS4 score (number of nodules multiplied by 1 + number of abscesses multiplied by 2 + number of draining tunnels multiplied by 4. Total score ≤3: mild disease, 4–10: moderate disease, 11 ≥ severe disease). HiSCR (achievement defined as ≥50% reduction in inflammatory lesion count and no increase in abscesses or fistulas compared to baseline), T0 (Time zero), Ts (Time of switch), T3 (Time at 3 months), T6 (Time at 6 months), T9 (Time at 9 months), T12 (Time at 12 months).

Abbreviations: BMI, body mass index; HS, Hidradenitis suppurativa; SD, standard deviation.

## CONFLICT OF INTEREST

The authors have no conflict of interest.

## ETHICS STATEMENT

The patients in this article have given written informed consent to publication of their case details.

## Data Availability

The data that support the findings of this study are available from the corresponding author upon reasonable request.
